# A Diagnostic Odyssey: From Pulmonic to Prosthetic Valve Endocarditis

**DOI:** 10.7759/cureus.106749

**Published:** 2026-04-09

**Authors:** Mustafa Arain, Taysir Al Janabi, Taha Abdul Rehman, Umair Tariq

**Affiliations:** 1 Internal Medicine, Wellspan York Hospital, York, USA; 2 Cardiology, Wellspan York Hospital, York, USA

**Keywords:** double valve infective endocarditis, infective endocarditis, prosthetic valve infective endocarditis, pulmonic valve endocarditis, viridans group streptococci

## Abstract

Infective endocarditis (IE) carries substantial morbidity and mortality despite appropriate antibiotic therapy and surgery. Right-sided IE is uncommon, and isolated pulmonic valve involvement is particularly rare. Echocardiography, transthoracic (TTE) and especially transesophageal (TEE), remains the cornerstone of diagnosis, although sensitivity may be limited, particularly in prosthetic valves.

We report a 65-year-old man with prior bioprosthetic aortic valve replacement who presented after outpatient blood cultures grew gram-positive cocci. He described six weeks of exertional dyspnea, drenching night sweats, and 20-lb unintentional weight loss without recreational substance use. Blood cultures speciated to *Streptococcus mitis* (oral group). Initial TEE revealed a mobile vegetation on the pulmonic valve consistent with IE. Despite vancomycin and ceftriaxone, his symptoms persisted. Three subsequent TEEs failed to show prosthetic valve involvement. A fourth TEE ultimately demonstrated a large vegetation on the bioprosthetic aortic valve. He underwent redo-sternotomy and repeat aortic valve replacement, followed by a six-week antibiotic course with gradual clinical recovery.

This case underscores the diagnostic complexity of IE with atypical presentations. Isolated pulmonic valve involvement without traditional risk factors is unusual and may resemble non-infectious processes. Prosthetic valve infection can emerge later in the disease course and evade detection on earlier imaging. In our patient, persistent but nonspecific symptoms, initially negative studies, and partial response to therapy obscured progression to prosthetic valve involvement. These features highlight the need for sustained clinical vigilance and timely repeat high-quality imaging when findings remain incongruent with the clinical course. Our report contributes to the limited literature on isolated pulmonic valve IE and its potential evolution to prosthetic valve infection.

## Introduction

Infective endocarditis (IE) has a high mortality rate despite treatment with appropriate antibiotic therapy and surgical intervention. The in-hospital mortality rate ranges from 15% to 20%, and the one-year mortality rate is approximately 40% [1]. The overall incidence of IE is 3-10 cases per 100,000 person-years, with a higher prevalence in older patients. Prosthetic valve endocarditis (PVE) represents 10%-30% of all cases of IE and carries a substantial in-hospital mortality of approximately 20%-40%, highlighting its severe clinical burden [2]. Right-sided IE is less common than left-sided IE, accounting for 5%-10% of all IE cases. Approximately 90% of right-sided IE cases involve the tricuspid valve, while isolated pulmonic valve endocarditis is rare, affecting only 1%-2% of all IE cases [3].

The low incidence of right-sided IE can be attributed to several factors, including a lower prevalence of congenital malformations and acquired valve abnormalities, differences in endothelial lining and vascularity, lower pressure gradients and jet velocities across right-sided valves, and reduced oxygen content of venous blood [4]. Risk factors for isolated pulmonic valve IE include congenital heart valve disease, central venous catheters, intracardiac devices, intravenous drug use, immunosuppression, and comorbidities such as HIV infection, diabetes mellitus, or chronic kidney disease. However, up to 28% of isolated pulmonic valve IE cases have been reported in patients with no identifiable risk factors [1].

Echocardiography, whether transthoracic (TTE) or transesophageal (TEE), is essential for the diagnosis and management of IE and should ideally be performed as soon as IE is suspected. If TTE findings are negative but clinical suspicion remains high, TEE is recommended, particularly when TTE image quality is poor. False-negative results may occur; the sensitivity of TTE for native and prosthetic valves has been reported to be approximately 70% and 50%, respectively, whereas TEE sensitivities are 96% and 92%, respectively [2]. The European Society of Cardiology (ESC) provides specific guidelines regarding the appropriate use of TTE and TEE in patients with suspected IE. Fever is the most common presenting symptom, occurring in approximately 90% of cases, while fatigue, chills, and night sweats are also common non-specific symptoms [5].

Here, we present a diagnostically challenging case of isolated pulmonic valve IE initially identified on three separate TEE examinations, with subsequent detection of prosthetic aortic valve IE on a fourth TEE. This case adds value to the limited literature describing isolated pulmonic valve IE with later development of prosthetic valve involvement.

## Case presentation

A 65-year-old man with a medical history significant for primary hyperparathyroidism, bicuspid aortic valve complicated by stenosis status post open aortic valve replacement, left atrial appendage ligation, atrial septal defect (ASD) status post primary closure, and benign prostatic hyperplasia (BPH) presented to the emergency department (ED) with rigors and night sweats. The patient reported four weeks of progressively worsening drenching night sweats, unintentional 20-pound weight loss, and exertional dyspnea that limited his ability to perform daily activities. He was an avid cyclist who routinely biked 200-300 miles per week. He denied intravenous drug use or recreational substance use.

Vital signs were within normal limits, and the initial physical examination was unremarkable. ED laboratory evaluation revealed mild normocytic anemia and mild hypercalcemia (10.4 mg/dL), which was stable compared to prior measurements. Inflammatory markers were elevated, with an erythrocyte sedimentation rate (ESR) of 45 mm/hour and C-reactive protein (CRP) of 74.3 mg/dL. D-dimer was elevated at 1.88 mg/L. Electrocardiogram demonstrated an atrial ectopic rhythm, and chest computed tomography angiography (CTA) was unremarkable.

Cardiology was consulted in the ED and recommended Lyme serology, outpatient TTE, and outpatient cardiology follow-up. Lyme testing returned negative. The first TTE demonstrated left ventricular hypertrophy with preserved ejection fraction (55%-60%) and a normally functioning bioprosthetic aortic valve, with no evidence of vegetations. The cardiology team advised age- and gender-appropriate malignancy screening.

The patient subsequently followed up with urology for an elevated prostate-specific antigen (PSA), which was felt unlikely to explain his symptoms. A CT scan of the abdomen and pelvis with contrast ordered by gastroenterology revealed mild splenomegaly without acute intra-abdominal pathology. Due to persistent anemia and hypercalcemia, the patient’s primary care physician ordered additional testing, including inflammatory markers, parathyroid hormone (PTH), parathyroid hormone-related peptide (PTHrP), serum protein electrophoresis (SPEP), and urine protein electrophoresis (UPEP). ESR and CRP remained elevated at 55 mm/hour and 88.6 mg/dL, respectively, while PTH, PTHrP, SPEP, and UPEP were unremarkable. The patient was referred to hematology-oncology for evaluation of a possible underlying malignancy. Blood cultures obtained during this evaluation grew gram-positive cocci in pairs, prompting referral back to the ED.

**Figure 1:**
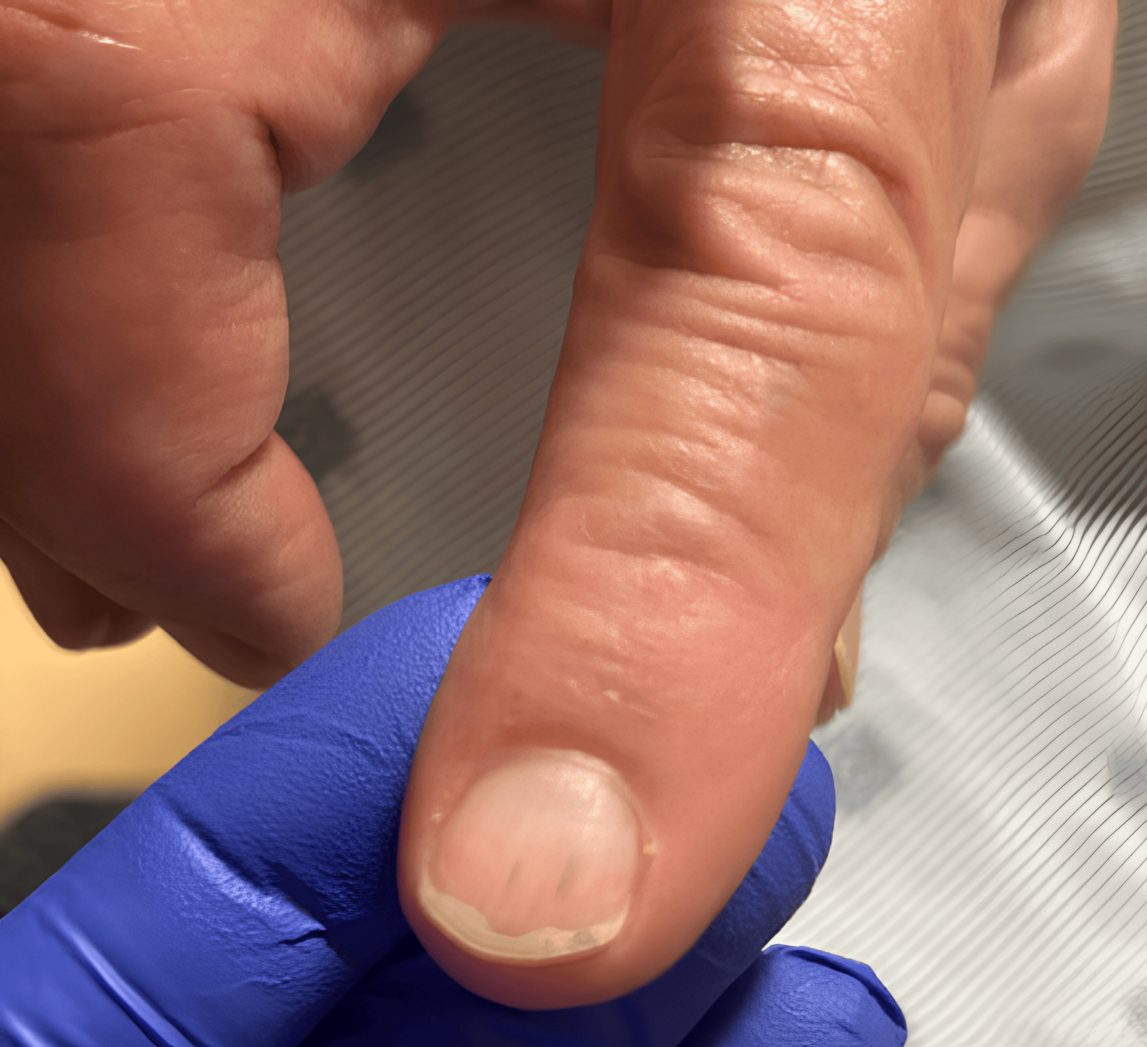
Splinter hemorrhage of the index finger.

On re-presentation, vital signs remained stable. Physical examination was notable for conjunctival pallor, splenomegaly, and a splinter hemorrhage of a fingernail (Figure 1). Laboratory studies demonstrated a normal lactate (1.1 mmol/L), normal procalcitonin (0.17 ng/mL), non-reactive HIV testing, and an unremarkable urine culture.

The patient’s symptoms initially improved, and repeat blood cultures showed no growth. He was scheduled for discharge on six weeks of intravenous antibiotics via a peripherally inserted central catheter (PICC), with plans for interval echocardiographic reassessment. However, before discharge, his symptoms worsened. Vancomycin was reintroduced, though repeat blood cultures remained negative. CTA of the chest showed no pulmonary embolism or septic emboli.

**Figure 2:**
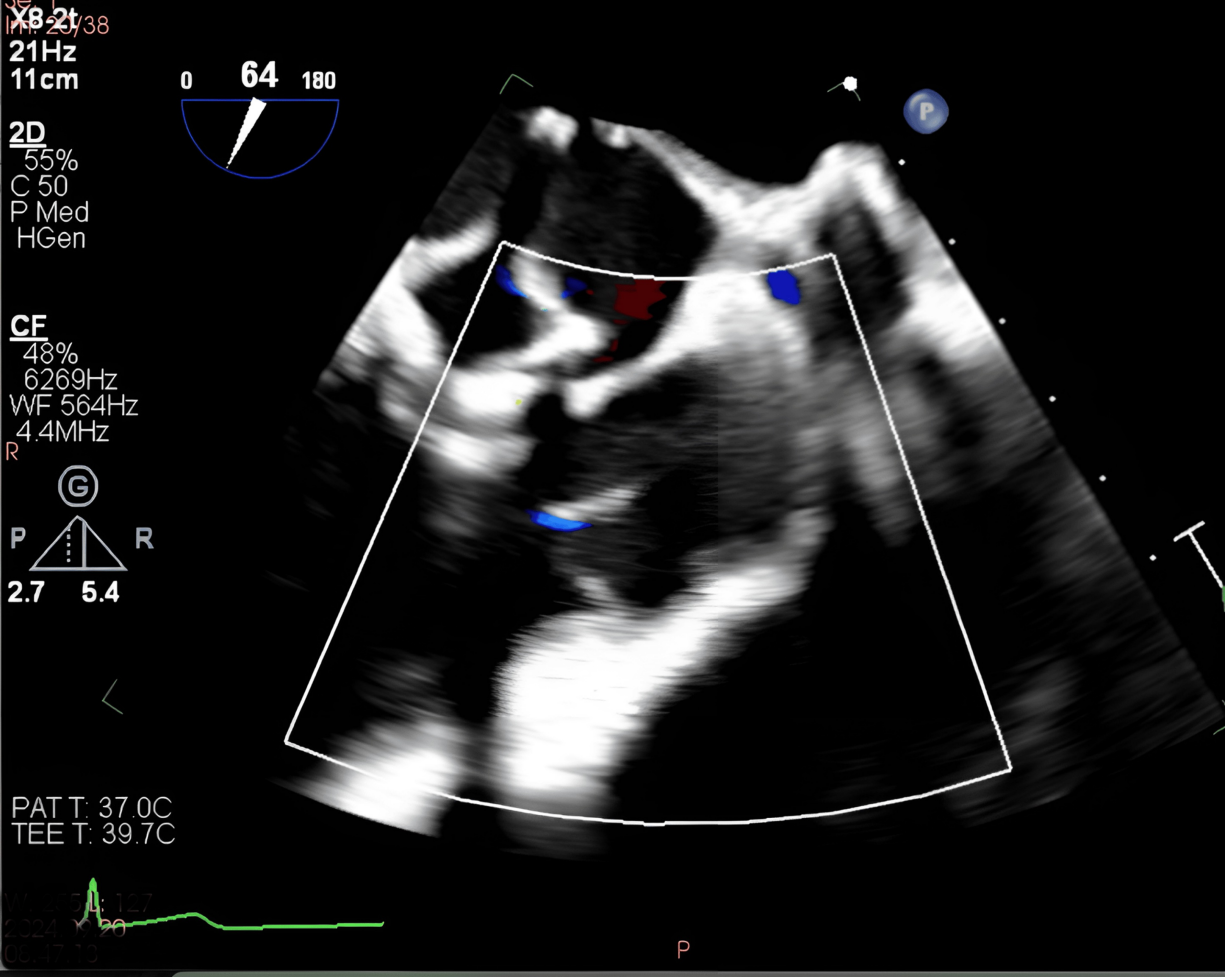
TEE showing pulmonic valve vegetation. TEE, transesophageal echocardiography

The patient was admitted and empirically started on vancomycin and cefepime, later narrowed to ceftriaxone due to concern for prosthetic valve IE. A TEE was obtained due to persistent high clinical suspicion despite a previously negative TTE. This TEE revealed a 0.6 × 0.7 cm mobile echodensity on the pulmonary arterial side of the pulmonic valve, consistent with vegetation (Figure 2). Blood cultures speciated as *Streptococcus mitis* (viridans group), and vancomycin was discontinued based on susceptibility results.

A second TEE demonstrated persistence of the pulmonic valve vegetation without significant interval change. Additionally, a repeat TTE obtained during this hospitalization again demonstrated normal prosthetic aortic valve function with no visible vegetations, reinforcing the absence of left-sided involvement at that time. Given persistent symptoms, non-infectious etiologies were considered, and a tagged white blood cell scan was obtained. The WBC scan later demonstrated normal radiotracer distribution. The patient’s symptoms again improved, inflammatory markers normalized, and he was discharged on vancomycin and ceftriaxone.

Four days after discharge, the patient returned to the ED with recurrent drenching night sweats, rigors, and headaches. ESR and CRP were again elevated. Brain imaging was unremarkable. A third TEE demonstrated a large mobile echodensity on the tip of the PICC line protruding into the right atrium, with no definitive evidence of prosthetic aortic or pulmonic valve infection. The PICC line was removed due to thrombosis. Despite extensive evaluation, including fluorodeoxyglucose positron emission tomography (FDG-PET) imaging, no clear evidence of active IE was identified.

**Figure 3:**
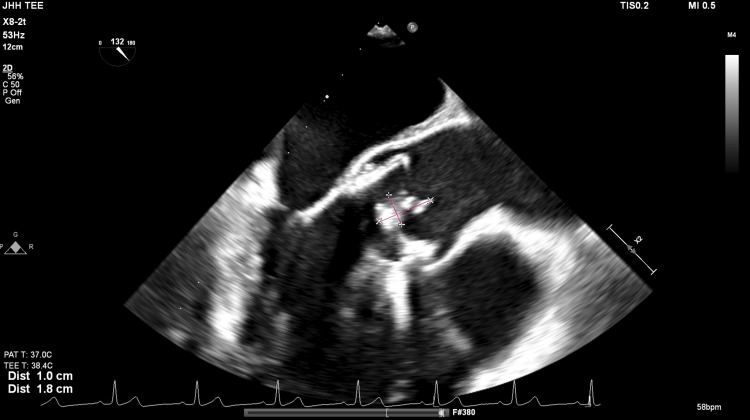
TEE showing 1.8 × 1.0 cm mobile vegetation on the bioprosthetic aortic valve. TEE, transesophageal echocardiography

**Figure 4:**
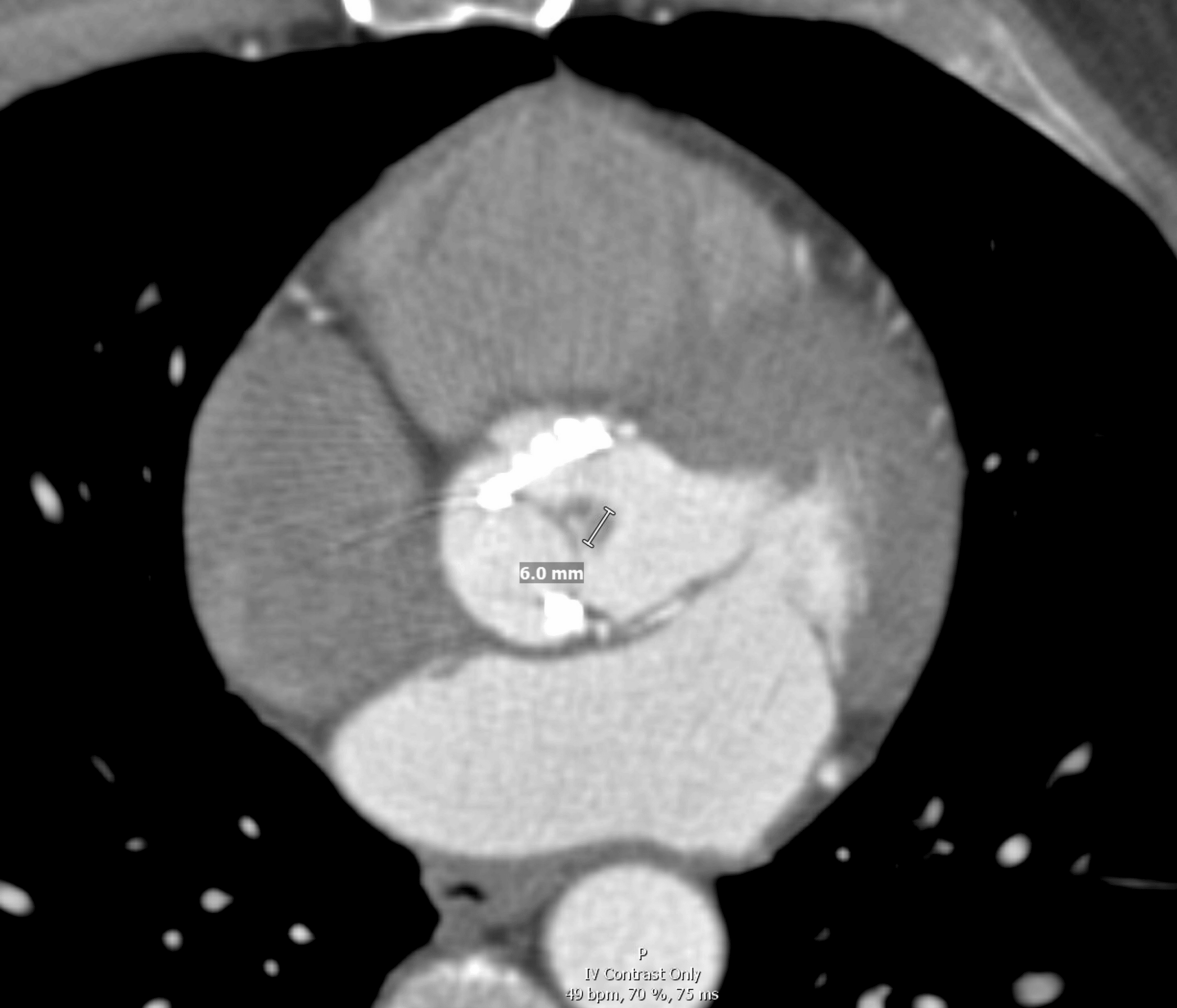
Coronary computed tomography angiography (CTA) showing a 6 mm vegetation on the prosthetic valve.

Ten days later, following another recurrence of symptoms, a second TTE at a tertiary care center again failed to demonstrate vegetations or prosthetic valve dysfunction. However, repeat (fourth) TEE at that time revealed a 1.8 cm × 1.0 cm mobile vegetation on the bioprosthetic aortic valve (Figure 3), as well as a thin filamentous lesion on the pulmonic valve. Coronary CTA confirmed a 6-mm vegetation on the prosthetic valve (Figure 4), and the patient underwent redo sternotomy and aortic valve replacement. He completed six weeks of postoperative antimicrobial therapy, with gradual clinical improvement and near-complete return to baseline functional status at three months.

## Discussion

Our patient presented with the non-specific symptoms of IE. However, his imaging and microbiological studies were consistent with IE. Our patient’s blood culture grew *S. mitis*, part of the viridans streptococci group - a well-established cause of IE. Notably, *S. mitis* possesses a high capacity for biofilm formation on prosthetic materials, including bioprosthetic valve surfaces, which may facilitate late seeding and persistent infection even after initial antibiotic therapy [6]. This biofilm-forming ability provides a plausible mechanistic link between the initial bacteremia and the delayed emergence of prosthetic valve vegetation in our patient. Additionally, our patient had a bioprosthetic aortic valve replacement, which put him at a higher risk for prosthetic valve IE compared to native valve IE. However, his repeated TEEs showed evidence of isolated pulmonic IE. The patient did not report any intravenous drug use or any other predisposing factors known for right-sided IE.

It was challenging to visualize vegetation on the pulmonic valve during the initial TTE; however, it was noted on TEE. The anatomic location of the pulmonic valve makes it hard to visualize on the echocardiogram. While TEE is more sensitive than TTE in detecting vegetation, both TTE and TEE have a specificity of 90% [2]. If the initial echocardiogram is non-diagnostic and there is high suspicion of IE, then a repeat echocardiogram a few days later is recommended [7]. TEE sensitivity should increase with repeated examination. However, our patient had three TEEs that did not detect the prosthetic valve IE. Additionally, the mobile echo density mass on the pulmonic valve of our patient might be an incidental finding of no clinical significance, as small mobile echogenic masses upon the cardiac surfaces are not uncommon; they may reflect small areas of valvular degeneration in the absence of infection [5]. Moreover, our patient had an intracardiac device, which might introduce an artifact and was reported as pulmonic valve vegetation on TEE.

A notable finding on the third TEE was a mobile echodensity on the tip of the PICC line protruding into the right atrium. After multidisciplinary discussion, our clinical consensus was that the PICC line most likely represented a secondary site of colonization rather than the primary nidus perpetuating bacteremia [8]. This conclusion was supported by several observations: (1) the patient’s bacteremia with *S. mitis* preceded PICC line placement, (2) symptoms and inflammatory markers initially improved with antibiotics before the PICC line was placed, and (3) removal of the line alone did not lead to sustained clinical resolution. Instead, we believe the persistent bacteremia from the initially unrecognized prosthetic valve infection seeded the PICC line secondarily. This distinction is important because it reinforces that device‑associated echodensities in IE patients should prompt evaluation for a possible underlying cardiac source rather than assuming it to be the primary focus.

Our case might have started with pulmonic valve IE, which initially responded to ceftriaxone and vancomycin. However, later in the course, prosthetic valve IE developed secondary to bacteremia seeding into the valves, which could explain why the three initial TEEs were negative for prosthetic valve vegetation. FDG-PET can be used to evaluate prosthetic valve IE in cases where there is an intracardiac device or concern for an artifact; FDG-PET has emerged as an adjunct diagnostic modality with a sensitivity of 93% for prosthetic valve IE [5,9]. However, this diagnostic modality did not show clear evidence of active IE in our patient. It is hard to discern when the prosthetic valve IE developed during the patient’s clinical course.

Regarding the tagged white blood cell (WBC) scan performed during the diagnostic workup, it demonstrated normal radiotracer distribution. While WBC scintigraphy is sometimes used as an adjunctive tool, echocardiography remains the central modality for diagnosis of IE, and a negative WBC scan should not be used to exclude IE when clinical suspicion persists [10].

Additionally, unlike our case, single-valve IE accounts for the majority of reported cases. In a case series of 77 patients with IE, 18% had multi-valvular involvement, primarily affecting the mitral and aortic valves [11]. Another study reported double-valve IE in 15.8% out of 280 IE cases, mainly affecting aortic and mitral valves [12].

As in our case, IE can present with very non-specific symptoms, mimicking malignancy. These non-specific symptoms can result in a delay in diagnosis, especially in individuals who do not have risk factors.

Limitations

Several limitations of this case warrant acknowledgment. First, the nationwide shortage of blood culture bottles at the time of the patient’s initial presentation delayed microbiological diagnosis, as cultures were not obtained on the first encounter [13]. This real‑world systemic constraint likely contributed to the prolonged diagnostic odyssey. Second, while multiple TEE studies were performed, the optimal timing and frequency of repeat imaging in suspected PVE remain undefined, and our approach was necessarily individualized. Third, FDG‑PET did not reveal active IE despite later confirmation of prosthetic valve vegetation, highlighting that a negative PET study does not exclude PVE, particularly when antibiotic therapy has been administered. Fourth, the tagged WBC scan was negative, which aligns with published data showing low sensitivity for valvular vegetations [10]. Finally, this single case limits generalizability, though the detailed chronology provides valuable lessons for clinical practice.

## Conclusions

This case adds to the limited literature on double-valve IE involving the pulmonic and prosthetic aortic valves in a patient without traditional right-sided IE risk factors. The non-specific clinical presentation and delayed radiographic detection emphasize the need for persistent diagnostic vigilance and repeated imaging when suspicion remains high.
